# Dose-volume relationships of planned versus estimated delivered radiation doses to pelvic organs at risk and side effects in patients treated with salvage radiotherapy for recurrent prostate cancer

**DOI:** 10.1016/j.tipsro.2023.100231

**Published:** 2023-12-14

**Authors:** Vilberg Jóhannesson, Adalsteinn Gunnlaugsson, Per Nilsson, Patrik Brynolfsson, Elisabeth Kjellén, Elinore Wieslander

**Affiliations:** a Radiation Physics, Department of Hematology, Oncology and Radiation Physics, Skåne University Hospital, Sweden; b Department of Hematology, Oncology and Radiation Physics, Skåne University Hospital, Lund, Sweden; c Lund University, Faculty of Medicine, Department of Clinical Sciences Lund, Radiation Physics, Lund, Sweden; d Lund University, Faculty of Medicine, Department of Clinical Sciences Lund, Oncology and Pathology, Lund, Sweden

**Keywords:** Organs at risk, Prostate cancer, Volumetric-modulated arc therapy, Estimated delivered dose, Salvage radiotherapy

## Abstract

**Purpose:**

To investigate estimated delivered dose distributions using weekly cone-beam computed tomography (CBCT) scans for pelvic organs at risk (OARs) in salvage radiotherapy (SRT) after radical prostatectomy. Furthermore, to compare them with the originally planned dose distributions and analyse associations with gastrointestinal (GI) and genitourinary (GU) side effects.

**Methods:**

This study is part of a phase II trial involving SRT for recurrent prostate cancer. Treatment was personalised based on PSA response during SRT, classifying patients as PSA responders or non-responders. Estimated radiation dose distributions were obtained using deformable image registration from weekly CBCT scans. GI and GU toxicities were assessed using the RTOG toxicity scale, while patient-reported symptoms were monitored through self-assessment questionnaires.

**Results:**

The study included 100 patients, with similar treatment-related side effects observed in both responders and non-responders. Differences in dose-volume metrics between the planned and estimated delivered doses for the examined OARs were mostly modest, although generally statistically significant. We identified statistically significant associations between QUANTEC-recommended dose-volume constraints and acute bowel toxicity, as well as late urinary patient-reported symptoms, for both the estimated delivered and planned dose distributions.

**Conclusion:**

We found small but statistically significant differences between estimated delivered and planned doses to OARs. These differences showed trends toward improved associations for estimated delivered dose distributions with side effects. Enhanced registration methods and imaging techniques could potentially further enhance the assessment of truly delivered doses and yield more reliable dose-volume constraints for future therapies.

## Highlights

• Evaluation of delivered dose in prostate salvage radiotherapy based on CBCT.
• Deformable CT/CBCT registration evaluated with Dice coefficient showed robust performance.
• Differences, although modest, were observed between planned and estimated delivered dose.
• Trend towards a better association between dose and side effects for delivered dose.

## Introduction

Pelvic radiotherapy (RT) poses challenges due to the mobile anatomy caused by variations in, for example, urinary bladder, rectal and bowel filling. Image-guided radiotherapy (IGRT) enables adequate dose coverage to the target volume. However, day-to-day variations in organ volumes and location are challenging. Treatment planning guidelines based on dose-volume histogram (DVH) recommendations rely on static planned dose distributions before the start of RT and do not consider these daily variations.

One major factor in achieving or violating dose constraints for the urinary bladder is the difference between planned and actual bladder volume [1], [2], [3]. Variations in urinary bladder dose metrics of up to 15–20 % have been observed for individual patients, with variations in bladder volume ranging from 16 % to 58 % on daily cone-beam computed tomography (CBCT) images [4]. It has also been suggested that radiation dose to bladder sub-volumes, such as the urethra, the bladder wall and the bladder trigone [5], [6], [7], [8], [9], [10], [11], [12], has a stronger association with toxicity than the dose to the whole organ [13]. Previous investigations have also shown variations in the delivered rectum DVHs, which might deviate considerably from the planned dose distributions [14], [15].

The estimated delivered dose to organs at risk (OAR) and targets during the entire treatment course, can be estimated based on CBCT images [16], [17] acquired at treatment. This can be accomplished by transferring the treatment plan to each CBCT image series, based on rigid registration, and summarising DVH metrics to represent the entire treatment [4]. Another approach is to use deformable image registration (DIR)[18], where deformation fields based on registration between the planning CT and each CBCT are applied to either the dose calculated on each CBCT or the original dose distribution [15], [19].

The primary aim of the present study was to compare planned and estimated delivered dose distributions in pelvic OARs for the patients enrolled in a salvage radiotherapy (SRT) study for prostate cancer (PROPER I). The secondary aim was to determine the potential association between dose-volume metrics for these dose distributions and gastrointestinal (GI) as well as genitourinary (GU) side effects.

## Materials and methods

### The PROPER I trial

The present study is part of the prospective clinical trial, PROPER I (NTC02699424), which enrolled patients between March 2016 and December 2019. The PROPER I trial is a single-centre, open-label, phase II trial including patients with biochemical recurrence after prostatectomy, conducted at Skåne University Hospital in Sweden [20]. The trial was approved by the Regional Ethics Review Board in Lund (Ref. No. 2015/431). The SRT was personalised, and patients were classified as responders or non-responders according to PSA response after five weeks (50 Gy/25 fractions (fr)) of SRT to the prostate bed based on PSA measurements before and during SRT. Responders continued SRT to the prostate bed to a total dose of 70 Gy/35 fr. For non-responders, SRT was adapted to include pelvic lymph nodes to 50 Gy/25 fr while keeping 70 Gy/35 fr to the prostate bed. The clinical target volume to planning target volume margin was 10 mm and 8 mm for the prostate bed and the pelvic lymph nodes, respectively. Macroscopic disease as diagnosed with PSMA-PET was treated with simultaneous integrated boost (lymph node metastases and/or local recurrence). This approach showed a high tumour control rate among non-responders. The concept is now being evaluated in a phase III trial, PROPER II (NCT04858889).

All patients in the PROPER I trial were treated with volumetric modulated arc therapy (VMAT), and the treatment planning was carried out in Eclipse versions 13.6 and 15.6 (Varian Medical Systems, Palo Alto, CA, USA) with the anisotropic analytical algorithm versions 10.0.28 and 13.6.23. The adaptation for non-responders to include pelvic lymph nodes was made with a sequential plan-on-plan VMAT technique, which has been previously reported [21]. Dose volume objectives from the PROPER I trial are presented in Supplementary Table 1. All patients received written information regarding bowel preparations (starting with stool bulking agents at least two weeks before the CT scan and continuing throughout the entire treatment period) and bladder preparations (emptying the bladder one hour before the CT scan and before each treatment for a comfortably filled bladder).

### Image guidance

Image guidance during treatment was based on CBCT images with automatic bone matching, and manual adjustment was carried out if required. The fixed scan length of the CBCT was 16 cm. A No Action Level protocol was used for the first three fractions, along with weekly imaging.

### Organs at risk delineation

OARs were delineated on weekly CBCT images by one senior dosimetrist. The rectum was delineated inferiorly from the lowest level of the ischial tuberosities to the level where the rectum loses its round shape in the axial plane and connects anteriorly with the sigmoid [22]. The bladder was delineated from its base to the dome. The most caudal slice of the bladder was kept the same in all CBCT as in the planning CT due to difficulty in defining the caudal limit of the bladder's on the CBCTs. Bladder trigone was delineated as a sub-volume of the bladder wall (created by adding an inner margin of 5 mm to the contoured bladder) ranging between the right and left ureteral orifices and the urethral orifice (Supplementary Fig. 1). The anal canal was delineated as the distal 4 cm of the rectum (i.e., a sub-volume of the rectum).

### Deformable image registration

DICOM-RT data, including CBCT images and associated rigid registrations, were exported from Eclipse and imported into the MICE toolkit v. 2021.2.1 (NONPI Medical AB, Sweden https://www.micetoolkit.com) for DIR [18], [23] and extraction of dose-volume data. The DIR between the planning CT and CBCT (with the planning CT as the reference) was based on the imported rigid registration. The DIR settings were fine-tuned based on the rectum and bladder, which were separately outlined in the planning CT and CBCT images. The quality of the DIR was assessed using the volumetric Dice similarity coefficient (vDSC) for rectum and bladder, in combination with visual examination for cases with low vDSC values. The vDSC was evaluated quantitatively according to the recommendations of the American Association of Physicists in Medicine task group 132 (AAPM TG132) [24].

The DIR for each weekly CBCT registration was applied to the planned dose distribution to obtain an estimated dose distribution in the CT geometry. These estimates were then summed to represent the spatial delivered dose distribution received over the entire course of treatment.

### Data collection

Dose volume metrics V_xGy_ (i.e., the volume receiving xx Gy or more) according to Quantitative Analyses of Normal Tissue Effects in the Clinic collaboration (QUANTEC) [14], [25], [26] complemented with the mean dose (D_mean_) and the near maximum dose (D_2%_) were further analysed regarding dose distribution and side effects. In detail, V_50Gy_, V_60Gy_, V_65Gy_, V_70Gy_ and D_2%_ were used for the rectum/anal canal and V_65Gy_, V_70Gy_, D_2%_ and D_mean_ for bladder/trigone which are an extension of the dose volume criteria used in the trial (Supplementary Table 1).

Physician-reported urinary and bowel toxicity were evaluated according to the RTOG toxicity scale [27] at baseline, at the end of SRT, and at 3 and 12 months after the end of SRT. Acute toxicity was defined as the highest toxicity score at the end of RT or at the three-month follow-up. Twelve-month toxicity was used as a surrogate for late toxicity. We employed a dichotomized approach, categorizing both bowel and bladder toxicity into grade 0–1 and grade ≥ 2 toxicity, respectively.

Patient-reported outcomes were monitored with two self-assessment questionnaires from the European Organization for Research and Treatment of Cancer (EORTC), the general EORTC QLQ-C30 [28], and the prostate cancer-specific EORTC QLQ-PR25 [29] for urinary and bowel symptoms as well as sexual function (presented in an earlier publication [20]). QLQ-PR25 functional and symptom raw scores were linearly transformed to a 0–100 scale according to the EORTC scoring manuals. A change of 10 % or more from baseline was defined as a clinically meaningful difference in symptoms (Patient Reported Outcome Measures, PROM_10%_) [30], [31].

### Statistics

Median DVHs and interquartile ranges (IQR) were derived for each OAR. The Wilcoxon signed-rank test was employed to analyse significant differences between specified planned and estimated delivered dose-volume metrics.

We analysed the association between these dose-volume metrics and bowel and urinary RTOG grade ≥ 2 toxicity, and PROM_10%_ using univariable logistic regression.

Differences in the dose-volume metrics for patients with and without RTOG grade ≥ 2 toxicity, as well for patients with and without patient-reported changes in urinary/bowel symptoms, PROM_10%_, were tested using the Wilcoxon signed-rank test. P-values < 0.05 were considered statistically significant.

All statistical analyses were based on the study database as of March 03, 2022. The calculations were performed using MedCalc Statistical Software, version 20.106, and R, version 4.2.2.

## Results

Detailed patient characteristics have been presented earlier [20]. In brief, 100 patients were enrolled in the trial, of whom 97 were eligible for outcome analysis. The mean age of the patients was 67 years (range 51–80). The median PSA level at the start of SRT was 0.25 ng/mL (IQR 0.19–0.37). Thirty-four (35 %) were classified as responders, and 63 (65 %) as non-responders. The median follow-up time was 38 months (IQR 29–48) [21].

Treatment-related side effects were similar for responders and non-responders. There was a tendency towards increased physician-reported acute GI toxicity for non-responders compared to responders. The patient-reported diarrhoea score was significantly higher in the non-responder group, while no statistically significant differences were found in either doctor-reported or patient-reported late side effects.

### Planned vs. estimated delivered dose

**Fig. 1:**
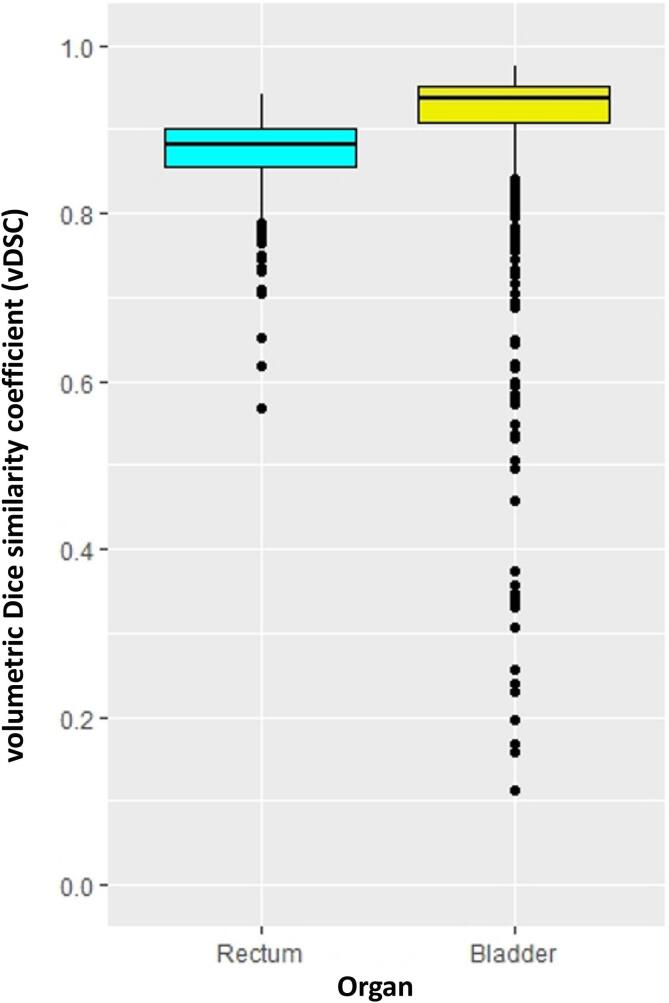
Box plots for the volumetric Dice similarity coefficient (vDSC) for the registration between the planning CT and the weekly CBCTs for the rectum and the bladder, respectively.

The registration between the planning CT and the weekly CBCTs was performed for seven CBCT studies for each responder and ten for each non-responder, resulting in 868 registrations. The median vDSC was 0.89 (IQR 0.86–0.90) and 0.93 (IQR 0.93–0.95) for the rectum and bladder, respectively (Fig. 1).

**Fig. 2:**
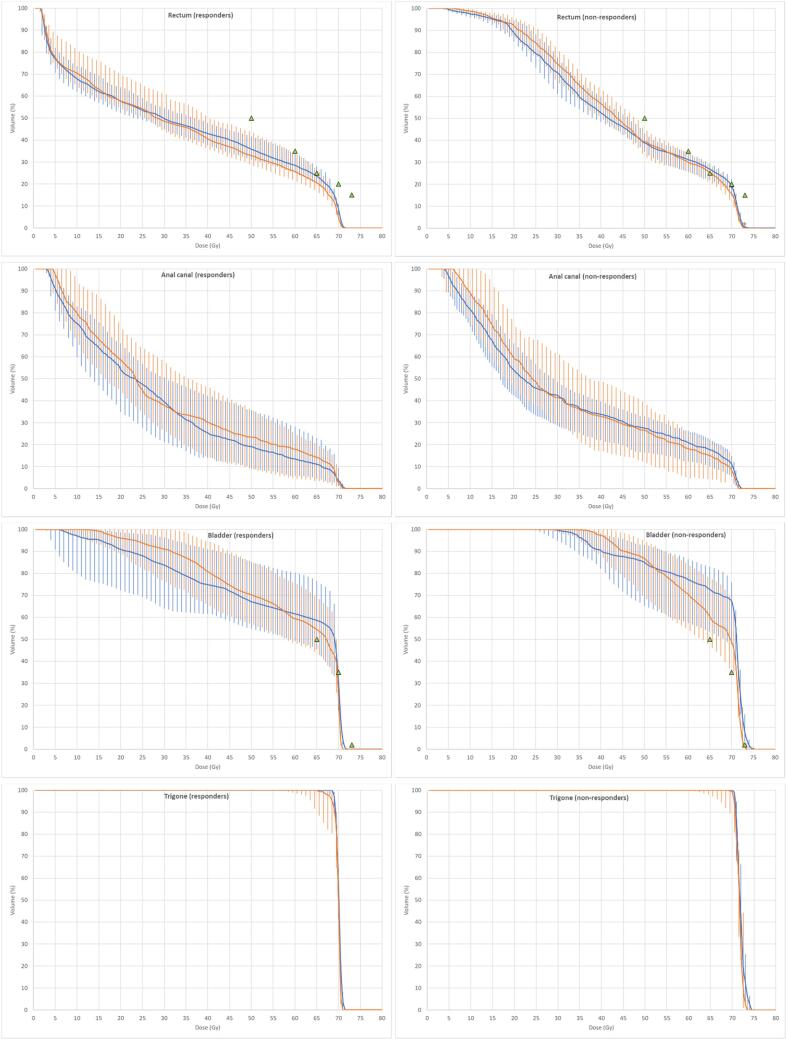
Median dose-volume histograms with interquartile ranges for the rectum, anal canal, bladder, and bladder trigone for planned dose (blue) and estimated delivered dose (brown) in responders and non-responders, respectively. Green triangles indicate evaluated QUANTEC criteria for the rectum and bladder. (For interpretation of the references to colour in this figure legend, the reader is referred to the web version of this article.)

Fig. 2 shows DVHs for each OAR separately for responders and non-responders with each graph including planned and estimated delivered doses. Data for the investigated dose-volume metrics (with V_x_ in relative units) are presented in Table 1. Although the median differences between the metrics for planned and estimated delivered dose distributions were rather small, they were statistically significant for all metrics for rectum, except for V_50Gy_ and D_mean_ for the urinary bladder.

**Table 1:** Comparison between selected planned and estimated delivered dose-volume metrics for the rectum, anal canal, bladder, and bladder trigone.

**All patients n 97Responders n 34**													**Non-responders n 63**									
**OARs** Median vol. (IQR)		**Planned dose/volume** Median 95 % CI		**Delivered dose/volume** Median 95 % CI		Median difference* Median 95 % CI		**pvalue**	**Planned dose/volume** Median 95 % CI		**Delivered dose/volume** Median 95 % CI		Median difference* Median 95 % CI		**p value**	**Planned dose/volume** Median 95 % CI		**Delivered dose/volume** Median 95 % CI		Median difference* Median 95 % CI		**p value**
**Rectum** 74.4cc (65.1–88.9)																						
**D_2%_**	**(Gy)**	**72.0**	71.6–72.3	**71.7**	71.1–71.9	**−0.4**	−0.5 — −0.3	**< 0.001**	**70.8**	70.7–70.9	**70.5**	70.3–70.7	**−0.4**	−0.5 — −0.2	**< 0.001**	**72.4**	72.3–72.6	**72.1**	71.9–72.3	**−0.5**	−0.6 — −0.3	**<0.001**
**V_50Gy_**	**(%)**	**38.2**	37.0–39.6	**37.7**	35.3–40.1	**0.1**	−0.9–1.1	0.79	**35.9**	31.9–39.0	**33.0**	29.8–39.0	**−0.5**	−2.6–1.2	0.59	**38.7**	37.6–39.4	**39.4**	36.6–41.9	**0.5**	−0.8–1.7	0.48
**V_60Gy_**	**(%)**	**30.7**	28.7–31.7	**28.7**	26.1–31.0	**−1.1**	−2.3 — −0.1	**0.025**	**28.6**	25.5–32.2	**25.8**	22.4–31.0	**−2.1**	−5.1 — −0.3	**0.020**	**31.2**	29.7–32.3	**30.0**	27.6–32.2	**−0.6**	−1.9–0.6	0.34
**V_65Gy_**	**(%)**	**26.3**	24.1–27.4	**23.2**	21.3–25.6	**−2.0**	−3.2 — −0.9	**< 0.001**	**23.6**	21.2–27.0	**20.6**	17.6–23.3	**−3.0**	−6.0 — −0.1	**0.0027**	**26.8**	25.3–28.3	**25.2**	22.6–26.2	**−1.4**	−2.8 — −0.3	**0.014**
**V_70Gy_**	**(%)**	**14.8**	13.1–17.2	**11.5**	9.5–14.7	**−2.4**	−3.4 — −1.7	**< 0.001**	**8.4**	6.4–10.1	**4.6**	3.2–7.2	**−2.2**	−3.6 — −1.2	**< 0.001**	**19.1**	16.5–20.2	**15.6**	13.5–18.0	**−2.5**	−3.9 — −1.6	**< 0.001**
**AnalCanal** 11.2 cc (9.1–15.0)																						
**D_2%_**	**(Gy)**	**71.2**	70.9–71.5	**70.9**	70.6–71.0	**−0.5**	−0.7 — −0.3	**< 0.001**	**70.7**	69.9–71.0	**70.1**	69.4–70.8	**−0.6**	−0.6 — −0.3	**< 0.001**	**71.6**	71.3–71.7	**71.2**	70.9–71.3	**−0.4**	−0.6 — −0.3	**< 0.001**
**V_50Gy_**	**(%)**	**23.9**	19.7–28.2	**25.4**	21.6–28.4	**0.3**	−1.1–1.9	0.71	**19.2**	15.7–27.1	**23.4**	10.0–29.4	**−1.2**	−3.8–1.9	0.45	**27.5**	21.6–29.5	**26.5**	21.9–30.3	**1.0**	−0.6–2.9	0.26
**V_60Gy_**	**(%)**	**18.4**	14.4–21.8	**18.2**	15.9–21.8	**−0.5**	−1.7–0.7	0.43	**13.6**	11.2–20.4	**17.9**	5.6–22.9	**−1.7**	−4.4–0.8	0.16	**21.0**	16.8–23.2	**18.2**	15.9–23.5	**0.1**	−1.2–1.4	0.88
**V_65Gy_**	**(%)**	**15.3**	12.1–18.6	**14.6**	11.9–17.5	**−0.9**	−2.0–0.1	0.096	**11.1**	7.4–17.0	**14.2**	4.5–17.9	**−1.8**	−4.0–0.6	0.14	**17.7**	14.3–19.4	**15.0**	11.9–18.8	**−0.6**	−1.7–0.5	0.32
**V_70Gy_**	**(%)**	**8.8**	6.1–10.9	**5.9**	4.8–8.4	**−1.3**	−2.0 — −0.7	**< 0.001**	**3.6**	1.8–8.0	**2.3**	0.5–6.1	**−0.9**	−2.0 — −0.1	**0.042**	**10.9**	8.6–12.9	**8.1**	5.1–9.7	**−1.5**	−2.4 — −0.7	**< 0.001**
**Bladder** 80.8cc (59.2–131.8)																						
**D_mean_**	**(Gy)**	**61.9**	58.4–64.5	**62.4**	59.2–63.6	**0.2**	−0.8–1.3	0.73	**55.2**	47.9–59.3	**56.9**	52.5–62.4	**1.6**	−1.2–5.2	0.24	**64.5**	61.8–66.2	**63.7**	61.1–66.0	**−0.3**	−1.2–0.7	0.60
**D_2%_**	**(Gy)**	**73.3**	72.9–73.6	**72.2**	72.0–72.6	**−0.8**	−0.9 — −0.7	**< 0.001**	**71.3**	71.1–71.4	**70.7**	70.6–70.9	**−0.5**	−0.6 — −0.4	**< 0.001**	**74.0**	73.5–74.3	**72.9**	72.6–73.1	**−0.9**	−1.1 — −0.8	**<0.001**
**V_65Gy_**	**(%)**	**69.1**	59.8–74.1	**57.4**	52.5–65.3	**−5.0**	−7.5 — −2.5	**< 0.001**	**58.7**	47.4–65.9	**54.5**	44.4–64.1	**−4.0**	−7.5–0.3	0.066	**72.7**	62.8–77.4	**60.4**	53.5–71.2	**−5.8**	−9.5 — −2.4	**0.001**
**V_70Gy_**	**(%)**	**48.2**	42.8–57.8	**38.8**	34.4–45.0	**−8.4**	−11.1 — −6.1	**< 0.001**	**29.7**	22.8–35.1	**22.2**	17.4–27.1	**−5.4**	−7.7 — −3.4	**< 0.001**	**67.4**	57.0–70.6	**48.8**	42.7–61.2	**−11.0**	−15.0 — −7.2	**< 0.001**
**Bladder Trigone** 8.6cc (7.3–10.5)																						
**D_mean_**	**(Gy)**	**71.2**	70.3–71.6	**70.8**	70.0–71.1	**−0.2**	−0.4 — −0.1	**< 0.001**	**70.0**	69.8–70.1	**69.8**	69.2–69.9	**−0.2**	−1.4–0.0	**0.0044**	**71.7**	71.5–72.1	**71.3**	71.1–71.7	**−0.2**	−0.4 — −0.1	**0.0018**
**D_2%_**	**(Gy)**	**73.1**	72.4–73.8	**72.4**	72.0–72.8	**−0.6**	−0.8 — −0.5	**< 0.001**	**71.3**	71.0–71.5	**70.9**	70.8–70.9	**−0.4**	−0.5 — −0.3	**< 0.001**	**74.1**	73.7–74.3	**73.1**	72.8–73.5	**−0.7**	−1.0 — −0.6	**< 0.001**
**V_65Gy_**	**(%)**	**100,0**	100.0–100.0	**100.0**	99.8–100.0	**−0.1**	−0.9–0.0	**0.0021**	**100.0**	100.0–100.0	**99.8**	97.3–100.0	**−0.6**	−6.0–0.0	**0.014**	**100.0**	100.0–100.0	**100.0**	100.0–100.0	**0.0**	−0.5–0.0	0.078
**V_70Gy_**	**(%)**	**98.3**	81.4–100.0	**86.7**	73.4–95.7	**−4.4**	−6.4 — −2.0	**< 0.001**	**55.0**	46.1–62.1	**47.3**	39.6–50.9	**−6.7**	−11.0 — −2.5	**0.0020**	**100.0**	100.0–100.0	**99.1**	95.2–99.9	**−3.0**	−5.7 — −0.6	**< 0.001**

*Hodges-Lehman median difference.

### Relationships between dose-volume metrics and gastrointestinal or genitourinary side effects

**Fig. 3:**
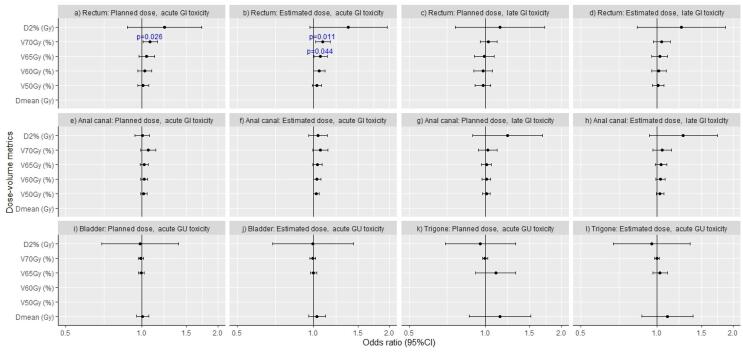
Odds ratios with 95 % confidence intervals for acute and late gastrointestinal (GI) or genitourinary (GU) grade ≥ 2 (according to RTOG) evaluated for the selected dose-volume metrics for both planned and estimated delivered dose distributions.

**Fig. 4:**
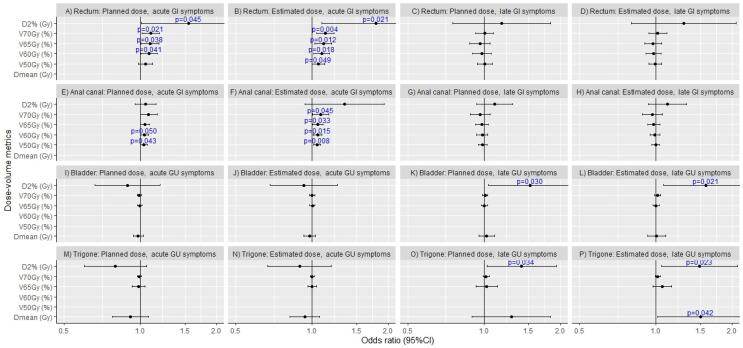
Odds ratios with 95% confidence intervals for Patient-Reported Outcome Measures (with a change of 10% or more from baseline, PROM_10%_) for bowel and urinary symptoms for the selected dose-volume metrics for planned and estimated delivered dose distributions.

As shown in Fig. 3, Fig. 4, we found statistically significant associations between several dose-volume metrics and side effects (RTOG grade ≥ 2 toxicity and PROM_10%_). Similar results were obtained for V_x_ in absolute units (data not shown). Due to the low incidence of late GU toxicity, logistic regression analysis was not applicable for that outcome parameter.

## Discussion

The present study investigated planned and estimated delivered dose distributions in prostate cancer patients treated with a novel adaptive SRT strategy for prostate cancer recurrence (the PROPER I trial). Our results showed statistically significant differences in dose-volume metrics between planned and estimated delivered doses for almost all metrics (rectum, anal canal, bladder, and bladder trigone) and these results were not affected by removing vDSC-outliers. However, these differences were modest in absolute terms, reflected in a similar association between dose-volume metrics and side effects for both planned and estimated delivered dose distributions that were well in line with QUANTEC recommendations (for example V_50Gy (%)_ to V_70Gy (%)_ for the rectum and D_2%_ for the bladder).

The metrics that demonstrated a stronger association with delivered doses were acute patient reported bowel toxicity as measured with the RTOG scale and PROM_10%_ (for rectum and anal canal) [32], and late urinary PROM_10%_ (bladder trigone). This indicates that estimating the dose to OARs on serial CBCTs could be more accurate for estimating the risk for side effects which is consistent, with previous studies [1], [2], [4], [15], [33].

The DIR technique used to align planning CT scans with weekly CBCT scans showed a good registration quality. The median vDSC for the rectum was 0.89 (IQR 0.86–0.90), indicating an approximate 89 % overlap between the rectal volumes on CBCT and planning CT. Similarly, the bladder showed a higher level of agreement, with a median vDSC of 0.93 (IQR 0.93–0.95), suggesting a 93 % overlap. These findings indicate accurate spatial correspondence between the planning CT and weekly CBCT scans for both the rectum and bladder, supporting the effectiveness of DIR to estimate delivered doses to specific organs [15], [34].

There are several limitations in our study. First, the limited cohort size and few events during the relatively short follow-up could explain the lack of correlation between dose-volume parameters and late toxicity. Furthermore, despite having a rather high vDSC for both bladder and rectum, we only had one CBCT per week and not for each treatment, which would have provided a more accurate estimate of the delivered dose. However, we consider that the multiple CBCTs gathered at regular intervals during the whole treatment course give a good estimation of possible changes in anatomy and dose geometry. Finally, the whole bowel bag was not included in the CBCTs, which prevented us from studying planned versus estimated delivered bowel doses and its association to diarrhoea.

Despite these limitations, our study contributes valuable information by identifying differences in dose-volume parameters between planned versus estimated delivered doses to OARs. However, as these differences are still modest derived with readily available methods for comparing planned versus estimated delivered doses, they can to some extent be explained by discrepancies in bowel and bladder preparation between planning CT and CBCTs. We find that current treatment planning methods for SRT for recurrent prostate cancer are still very robust, provided optimal treatment preparations are made. Our results stimulate further work to enable faster and more accurate estimations of the delivered dose during therapy for future treatment adaptations. Additionally, our findings contribute to generating better estimations of the correlation between dose distribution parameters and the risk for side effects.

## Conclusion

The differences between planned and estimated delivered dose distributions evaluated with weekly CBCTs, as presented in this work, were found to be small, however with statistically significant differences for QUANTEC recommended dose-volume constraints. Similarly, there was a trend towards a stronger association with side effects for some estimated rectal, bladder (and bladder trigone), and anal canal dose metrics as compared to planned doses. The results motivate development of future methods, preferably AI-based, to enhance precision and speed in registrations for more accurate, rapid, and clinically meaningful dose-volume evaluations. Such methods would enable automatic retrieval of dose-volume data to generate more accurate data during treatment. Until such methods are commercially available, traditional treatment planning for SRT with optimal bladder and rectal preparations are standard of care.

## Declaration of competing interest

The authors declare that they have no known competing financial interests or personal relationships that could have appeared to influence the work reported in this paper.
