# Supplementary material for: Dose-volume relationships of planned versus estimated delivered radiation doses to pelvic organs at risk and side effects in patients treated with salvage radiotherapy for recurrent prostate cancer
Source: Tech Innov Patient Support Radiat Oncol. 2023 Dec 14;29:100231. doi: 10.1016/j.tipsro.2023.100231 (PMC10772375; doi:10.1016/j.tipsro.2023.100231)
Supplement: Supplementary data 1 [file mmc1.docx]

| **Priority** | **Structure** | **Prescribed dose (dose/fraction)** | **Dose/volume objectives** |
| --- | --- | --- | --- |
| 1 | GTV-T | 70+ Gy (2.0+) | D_99%_≥ 76 Gy |
| 2 | CTV-T | 70+ Gy (2.0+) | D_98%_≥ 76 Gy |
| 3 | PTV-T | 70+ Gy (2.0+) | D_98%_≥ 74 Gy |
| 4 | CTV-P | 70 Gy (2.0) | D_99%_≥ 68 Gy |
| 5 | PTV-P | 70 Gy (2.0) | D_98%_ ≥ 66 Gy |
| 6 | GTV-Lmet | 60 Gy (2.4) | D_99%_≥ 58 Gy |
| 7 | CTV-Lmet | 60 Gy (2.4) | D_98%_≥ 58 Gy |
| 8 | PTV-Lmet | 60 Gy (2.4) | D_98%_≥ 57 Gy |
| 9 | Fixed bowel loop |  | V_50Gy_ < 17 cm^3^ |
|  |  |  | D_2%_≤ 60 Gy |
| 10 | Rectum |  | V_70Gy_< 20% |
| 11 | CTV-N | 50 Gy (2.0) | D_99%_ ≥ 47.5 Gy |
| 12 | PTV-N | 50 Gy (2.0) | D_99%_ ≥ 46.5 Gy |
| 13 | Femoral heads |  | D_max_≤ 55 Gy |
| 14 | BowelBag - PTV5mm |  | V_30Gy_< 300 cm^3^ |
|  |  |  | V_40Gy_ < 150 cm^3^ |
|  |  |  | V_45Gy_ < 100 cm^3^ |
|  |  |  | V_50Gy_ < 35 cm^3^ |
| 15 | Rectum |  | V_60Gy_< 35% |
| 16 | BODY |  | D_max_≤ 82 Gy |
| 17 | Bladder |  | D_Mean_≤ 62 Gy |

Supplementary Table 1

Dose volume objectives in the PROPER 1 trial.

**
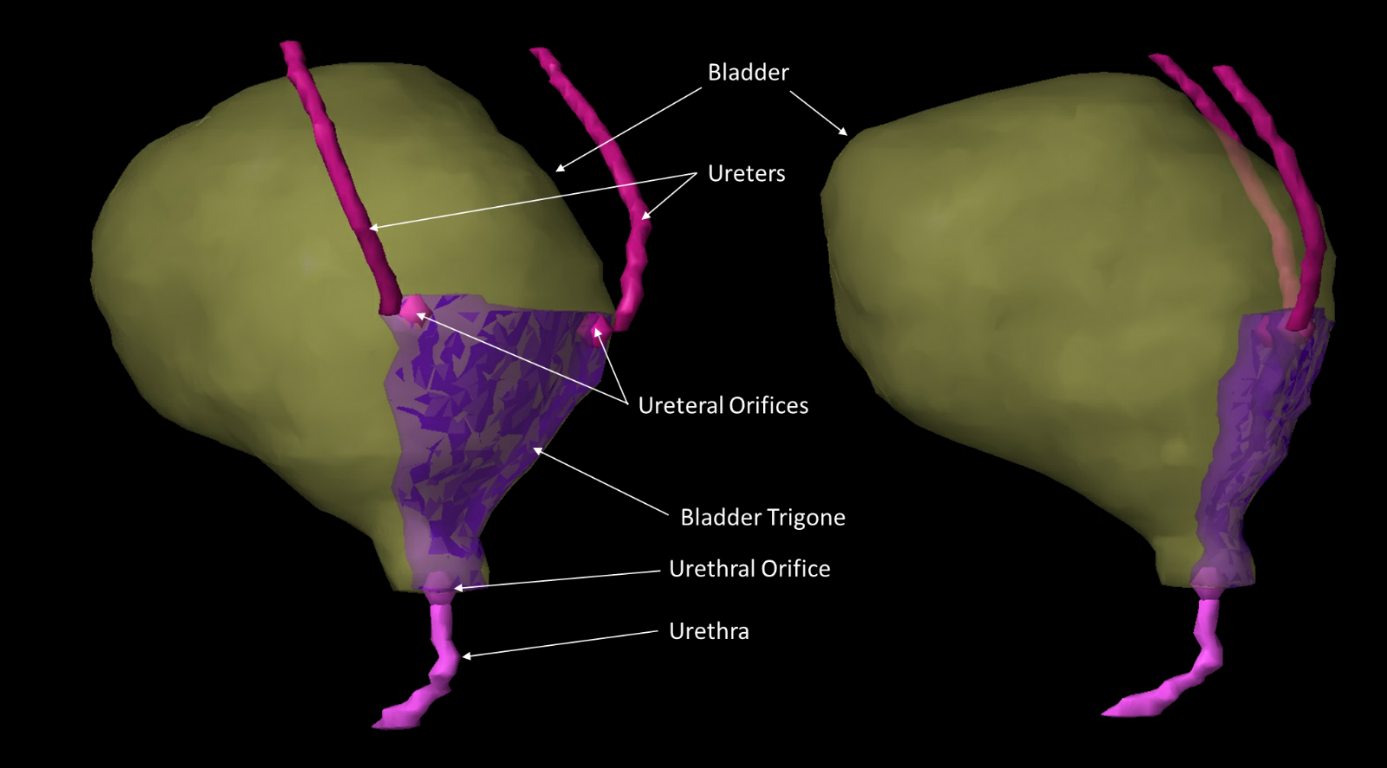
**

Supplementary Fig. 1

Images of the urinary bladder and bladder trigone from a posterior and lateral view.
